# Supplementary material for: Epidemiological Analysis of Diabetes-Related Hospitalization in Poland before and during the COVID-19 Pandemic, 2014–2020
Source: Int J Environ Res Public Health. 2022 Aug 14;19(16):10030. doi: 10.3390/ijerph191610030 (PMC9407838; doi:10.3390/ijerph191610030)
Supplement: Supplementary file 1 [file ijerph-19-10030-s001.zip › ijerph-1827668-supplementary.pdf]

Supplementary material

"Epidemiological analysis of diabetes-related hospitalization in Poland before and during the COVID-19 pandemic, 2014-2020"

Kuba Sękowski [1,*], Justyna Grudziąż-Sękowska [1], Paweł Goryński [2], Jarosław Pinkas [1] and Mateusz Jankowski [1]

[1] School of Public Health, Centre of Postgraduate Medical Education, 01-826 Warsaw, Poland
[2] Department of Population Health Monitoring and Analysis, National Institute of Public Health - National Institute of Hygiene, 00-791 Warsaw, Poland
* Correspondence kuba.sekowski@gmail.com

Supplementary Table S1. Diabetes-related hospitalizations in Poland between 2014-2019 – crude numbers

Type of diabetes mellitus	2014 n=75476						2015 n=74826						2016 n=76220						2017 n=74670					
	Overall		Males		Females		Overall		Males		Females		Overall		Males		Females		Overall		Males		Females	
	n	rate	n	rate	n	rate	n	rate	n	rate	n	rate	n	rate	n	rate	n	rate	n	rate	n	rate	n	rate
Overall	75476	196.2	38271	205.5	37205	187.3	74826	194.7	38390	206.4	36436	183.7	76220	198.3	39314	211.4	36906	186.0	74670	194.3	39018	209.9	35652	179.7
E10	30931	80.4	16610	89.2	14321	72.1	28890	75.2	15559	83.7	13331	67.2	30027	78.1	16274	87.5	13753	69.3	30351	79.0	16621	89.4	13730	69.2
E11	41275	107.3	19741	106.0	21534	108.4	42599	110.8	20849	112.1	21750	109.6	42821	111.4	21001	113.0	21820	110.0	40722	106.0	20220	108.7	20502	103.3
E13	1714	4.5	1026	5.5	688	3.5	1805	4.7	1089	5.9	716	3.6	1749	4.6	1084	5.8	665	3.4	1730	4.5	1071	5.8	659	3.3
E14	1556	4.0	894	4.8	662	3.3	1532	4.0	893	4.8	639	3.2	1623	4.2	955	5.1	668	3.4	1867	4.9	1106	5.9	761	3.8

Type of diabetes mellitus	2018 n=72188						2019 n=68906						2020 n=45159						Percentage difference 2019-2020					
	Overall		Males		Females		Overall		Males		Females		Overall		Males		Females		Overall		Males		Females	
	n	rate	n	rate	n	rate	n	rate	n	rate	n	rate	n	rate	n	rate	n	rate	n	rate	n	rate	n	rate
Overall	72188	187.9	38132	205.2	34056	171.7	68906	179.5	36464	196.4	32442	163.7	45159	118.0	24857	134.3	20302	102.7	-34.5	-34.2	-31.8	-31.6	-37.4	-37.2
E10	28584	74.4	15967	85.9	12617	63.6	28617	74.6	15852	85.4	12765	64.4	20282	53.0	11573	62.5	8709	44.1	-29.1	-28.9	-27.0	-26.7	-31.8	-31.6
E11	39904	103.9	19961	107.4	19943	100.6	38138	99.4	19322	104.1	18816	95.0	23568	61.6	12484	67.5	11084	56.1	-38.2	-38.0	-35.4	-35.2	-41.1	-40.9
E13	1663	4.3	1048	5.6	615	3.1	173	0.5	114	0.6	59	0.3	107	0.3	70	0.4	37	0.2	-38.2	-38.0	-38.6	-38.4	-37.3	-37.1
E14	2037	5.3	1156	6.2	881	4.4	1978	5.2	1176	6.3	802	4.0	1202	3.1	730	3.9	472	2.4	-39.2	-39.0	-37.9	-37.7	-41.1	-41.0

Abbreviation: rate - number of diabetes-related hospitalizations per 100.000 inhabitants

Supplementary Table S2. Demographic characteristics of patients hospitalized with type 1 diabetes in Poland between 2014-2019 – crude numbers and rates by age groups.

Age group	2014						2015						2016						2017					
	Overall n=30931		Males n=16610		Females n=14321		Overall n=28890		Males n=15559		Females n=13331		Overall n=30027		Males n=16274		Females n=13753		Overall n=30351		Males n=16621		Females n=13730	
	n	rate	n	rate	n	rate	n	rate	n	rate	n	rate	n	rate	n	rate	n	rate	n	rate	n	rate	n	rate
0-9	2147	54.3	1089	53.6	1058	54.9	1939	49.0	969	47.7	970	50.4	2276	57.5	1163	57.2	1113	57.8	2417	61.0	1247	61.3	1170	60.7
10-19	5136	133.6	2568	130.3	2568	137.0	4592	121.6	2305	119.1	2287	124.3	5300	142.0	2697	140.9	2603	143.1	6200	166.3	3151	164.8	3049	167.9
20-29	2135	39.2	1080	38.9	1055	39.4	2272	43.3	1244	46.6	1028	39.9	2322	45.9	1198	46.5	1124	45.3	2148	43.9	1113	44.7	1035	43.1
30-39	2465	39.0	1521	47.5	944	30.3	2550	40.2	1619	50.3	931	29.7	2506	39.6	1559	48.6	947	30.3	2443	38.8	1508	47.3	935	30.1
40-49	2587	52.2	1706	68.4	881	35.8	2577	50.9	1642	64.4	935	37.2	2503	48.1	1654	63.2	849	32.8	2616	49.0	1722	64.1	894	33.7
50-59	4311	79.7	2840	107.8	1471	53.1	3674	70.0	2377	92.8	1297	48.3	3516	69.1	2294	92.2	1222	47.0	3308	67.1	2188	90.7	1120	44.5
60-69	5501	118.5	3261	154.1	2240	88.7	5226	106.9	3013	135.0	2213	83.3	5441	108.3	3273	142.4	2168	79.5	5079	99.1	3154	134.3	1925	69.3
70-79	3918	164.9	1667	178.8	2251	155.9	3501	149.1	1535	165.9	1966	138.2	3561	147.2	1582	164.5	1979	135.8	3619	143.4	1675	165.4	1944	128.6
80+	2731	178.6	878	187.9	1853	174.5	2559	163.3	855	177.8	1704	156.9	2602	161.4	854	171.9	1748	156.7	2521	153.5	863	169.9	1658	146.1

Age group	2018						2019						2020						Percentage difference (2019-2020)					
	Overall n=28584		Males n=15967		Females n=12617		Overall n=28617		Males n=15852		Females n=12765		Overall n=20282		Males n=11573		Females n=8709		Overall		Males		Females	
	n	rate	n	rate	n	rate	n	rate	n	rate	n	rate	n	rate	n	rate	n	rate	n	rate	n	rate	n	rate
0-9	2303	58.7	1191	59.1	1112	58.3	2335	60.4	1233	62.0	1102	58.6	1775	46.6	920	47.0	855	46.1	-24.0	-22.9	-25.4	-24.3	-22.4	-21.3
10-19	5975	158.6	3057	158.3	2918	158.9	6411	167.8	3216	164.2	3195	171.5	4502	116.6	2297	116.1	2205	117.2	-29.8	-30.5	-28.6	-29.3	-31.0	-31.7
20-29	2000	42.4	1146	47.7	854	37.0	2061	45.4	1101	47.6	960	43.1	1245	28.5	694	31.1	551	25.7	-39.6	-37.3	-37.0	-34.5	-42.6	-40.4
30-39	2558	41.0	1570	49.7	988	32.1	2489	40.5	1566	50.3	923	30.4	1517	25.1	994	32.5	523	17.5	-39.1	-38.1	-36.5	-35.5	-43.3	-42.4
40-49	2335	42.6	1608	58.3	727	26.7	2419	42.9	1637	57.7	782	28.0	1695	29.4	1192	41.1	503	17.6	-29.9	-31.6	-27.2	-28.9	-35.7	-37.2
50-59	2942	61.5	1989	84.8	953	39.1	2862	61.3	1939	84.6	923	38.8	1940	42.1	1299	57.4	641	27.4	-32.2	-31.3	-33.0	-32.2	-30.6	-29.5
60-69	4713	90.8	2971	124.8	1742	62.0	4657	89.2	2826	117.8	1831	64.9	3450	66.5	2284	95.8	1166	41.6	-25.9	-25.4	-19.2	-18.7	-36.3	-35.9
70-79	3436	129.5	1617	150.5	1819	115.2	3260	116.7	1587	139.3	1673	101.2	2579	88.0	1317	109.7	1262	72.9	-20.9	-24.6	-17.0	-21.2	-24.6	-27.9
80+	2322	139.4	818	158.3	1504	130.9	2123	125.5	747	141.8	1376	118.1	1579	93.8	576	110.3	1003	86.3	-25.6	-25.3	-22.9	-22.2	-27.1	-26.9

Supplementary Table S3. Demographic characteristics of patients hospitalized with type 2 diabetes in Poland between 2014-2019 – crude numbers and rates by age groups.

Age group	2014						2015						2016						2017					
	Overall n=41275		Males n=19741		Females n=21534		Overall n=42599		Males n=20849		Females n=21750		Overall n=42821		Males n=21001		Females n=21820		Overall n=40722		Males n=20220		Females n=20502	
	n	rate	n	rate	n	rate	n	rate	n	rate	n	rate	n	rate	n	rate	n	rate	n	rate	n	rate	n	rate
0-9	14	0,4	7	0,3	7	0,4	15	0,4	7	0,3	8	0,4	33	0,8	20	1,0	13	0,7	37	0,9	19	0,9	18	0,9
10-19	120	3,1	61	3,1	59	3,1	107	2,8	57	2,9	50	2,7	121	3,2	56	2,9	65	3,6	124	3,3	52	2,7	72	4,0
20-29	267	4,9	168	6,1	99	3,7	288	5,5	164	6,1	124	4,8	278	5,5	156	6,1	122	4,9	307	6,3	184	7,4	123	5,1
30-39	806	12,8	559	17,5	247	7,9	905	14,3	618	19,2	287	9,2	962	15,2	707	22,0	255	8,2	921	14,6	651	20,4	270	8,7
40-49	2432	49,1	1751	70,2	681	27,7	2543	50,2	1790	70,2	753	29,9	2575	49,5	1859	71,0	716	27,7	2398	44,9	1691	62,9	707	26,6
50-59	7364	136,2	4611	175,0	2753	99,3	7243	138,1	4606	179,9	2637	98,2	6709	131,8	4257	171,1	2452	94,2	6025	122,3	3865	160,2	2160	85,9
60-69	11972	257,9	6375	301,2	5597	211,5	12995	265,8	7147	320,3	5848	220,1	12758	253,9	7079	308,1	5679	208,3	12235	238,6	6991	297,7	5244	188,7
70-79	10105	425,3	3788	406,4	6317	437,5	10167	433,1	4020	434,6	6147	432,1	10567	436,9	4291	446,3	6276	430,7	10324	409,1	4309	425,5	6015	398,0
80+	8195	536,0	2421	518,2	5774	543,8	8336	531,9	2440	507,4	5896	542,8	8818	547,1	2576	518,7	6242	559,7	8351	508,5	2458	484,0	5893	519,4

Age group	2018						2019						2020						Percentage difference (2019-2020)					
	Overall n=39904		Males n=19961		Females n=19943		Overall n=38138		Males n=19322		Females n=18816		Overall n=23568		Males n=12484		Females n=11084		Overall		Males		Females	
	n	rate	n	rate	n	rate	n	rate	n	rate	n	rate	n	rate	n	rate	n	rate	n	rate	n	rate	n	rate
0-9	37	0,9	14	0,7	23	1,2	44	1,1	18	0,9	26	1,4	40	1,0	22	1,1	18	1,0	-9,1	-7,8	22,2	24,0	-30,8	-29,8
10-19	125	3,3	73	3,8	52	2,8	152	4,0	66	3,4	86	4,6	117	3,0	54	2,7	63	3,3	-23,0	-23,8	-18,2	-19,0	-26,7	-27,5
20-29	304	6,5	170	7,1	134	5,8	281	6,2	156	6,7	125	5,6	183	4,2	109	4,9	74	3,5	-34,9	-32,4	-30,1	-27,4	-40,8	-38,5
30-39	949	15,2	683	21,6	266	8,6	981	16,0	688	22,1	293	9,7	606	10,0	444	14,5	162	5,4	-38,2	-37,2	-35,5	-34,4	-44,7	-43,8
40-49	2387	43,5	1732	62,8	655	24,1	2361	41,9	1715	60,5	646	23,1	1422	24,7	1057	36,4	365	12,7	-39,8	-41,2	-38,4	-39,8	-43,5	-44,9
50-59	5610	117,3	3630	154,8	1980	81,2	5043	108,0	3293	143,6	1750	73,6	3150	68,4	2163	95,5	987	42,2	-37,5	-36,7	-34,3	-33,5	-43,6	-42,7
60-69	11806	227,5	6774	284,6	5032	179,2	11188	214,4	6541	272,7	4647	164,7	6751	130,2	4122	173,0	2629	93,8	-39,7	-39,3	-37,0	-36,6	-43,4	-43,1
70-79	10399	391,9	4389	408,5	6010	380,6	10062	360,3	4397	385,9	5665	342,6	6530	222,8	3047	253,9	3483	201,3	-35,1	-38,1	-30,7	-34,2	-38,5	-41,2
80+	8287	497,5	2496	483,1	5791	504,0	8026	474,4	2448	464,8	5578	478,8	4769	283,2	1466	280,7	3303	284,3	-40,6	-40,3	-40,1	-39,6	-40,8	-40,6
